# Polarity-specific transcranial direct current stimulation disrupts auditory pitch learning

**DOI:** 10.3389/fnins.2015.00174

**Published:** 2015-05-18

**Authors:** Reiko Matsushita, Jamila Andoh, Robert J. Zatorre

**Affiliations:** ^1^Cognitive Neuroscience Unit, Montreal Neurological Institute, McGill UniversityMontreal, QC, Canada; ^2^International Laboratory for Brain, Music, and Sound ResearchMontreal, QC, Canada; ^3^Centre for Research on Brain, Language, and MusicMontreal, QC, Canada; ^4^Department of Cognitive and Clinical Neuroscience, Central Institute of Mental Health MannheimMannheim, Germany

**Keywords:** transcranial direct current stimulation, auditory pitch learning, pitch discrimination, consolidation, anodal tDCS

## Abstract

Transcranial direct current stimulation (tDCS) is attracting increasing interest because of its potential for therapeutic use. While its effects have been investigated mainly with motor and visual tasks, less is known in the auditory domain. Past tDCS studies with auditory tasks demonstrated various behavioral outcomes, possibly due to differences in stimulation parameters, task-induced brain activity, or task measurements used in each study. Further research, using well-validated tasks is therefore required for clarification of behavioral effects of tDCS on the auditory system. Here, we took advantage of findings from a prior functional magnetic resonance imaging study, which demonstrated that the right auditory cortex is modulated during fine-grained pitch learning of microtonal melodic patterns. Targeting the right auditory cortex with tDCS using this same task thus allowed us to test the hypothesis that this region is causally involved in pitch learning. Participants in the current study were trained for 3 days while we measured pitch discrimination thresholds using microtonal melodies on each day using a psychophysical staircase procedure. We administered anodal, cathodal, or sham tDCS to three groups of participants over the right auditory cortex on the second day of training during performance of the task. Both the sham and the cathodal groups showed the expected significant learning effect (decreased pitch threshold) over the 3 days of training; in contrast we observed a blocking effect of anodal tDCS on auditory pitch learning, such that this group showed no significant change in thresholds over the 3 days. The results support a causal role for the right auditory cortex in pitch discrimination learning.

## Introduction

Transcranial direct current stimulation (tDCS) is a non-invasive technique to modulate cortical excitability (Nitsche et al., 2008; Jacobson et al., 2012). The neurophysiological basis of this technique comes from animal studies using direct current stimulation, which have shown that anodal stimulation causes depolarization and cathodal stimulation causes hyperpolarization of neurons, leading to an alteration of spontaneous neural activity (Bindman et al., 1964; Purpura and McMurtry, 1965) not only during the stimulation, but also for hours after the end of stimulation (Bindman et al., 1964). In humans, measurement of motor evoked potentials (MEP) revealed that anodal tDCS over motor cortex increases cortical excitability and cathodal tDCS decreases it (Nitsche and Paulus, 2000; Nitsche et al., 2003a) and that the effect can last for more than an hour after application of the current with conventional parameters (Nitsche and Paulus, 2001; Nitsche et al., 2003a). At the same time, the direction of excitatory and inhibitory effects does not depend only on the polarity, but also on neural morphology in the affected area (Radman et al., 2009) and current intensity (Batsikadze et al., 2013), and effects from anodal tDCS and cathodal tDCS are not necessarily opposite (Matsunaga et al., 2004). For the mechanism of the after-effect, pharmacological studies have suggested involvement of NMDA receptors and GABAergic system (Liebetanz et al., 2002; Nitsche et al., 2003b, 2004), and several magnetic resonance spectroscopy (MRS) studies have observed changes in GABA and glutamate concentrations after tDCS application (Stagg et al., 2009; Clark et al., 2011), suggesting LTP and LTD-like mechanisms.

At a behavioral level, the effect of tDCS has been observed in learning (motor: Nitsche et al., 2003c; Boggio et al., 2006; Reis et al., 2009; Stagg et al., 2011; Kaminski et al., 2013, visuomotor: Antal et al., 2004; Vollmann et al., 2013, language: Flöel et al., 2008), and in perception (tactile: Rogalewski et al., 2004, visual: Antal et al., 2001; Costa et al., 2012). In particular, tDCS-induced effects seem to be relevant to learning as supported by physiological evidence obtained in an MRS study (Floyer-Lea et al., 2006); a reduction of GABA along with performance improvement has also been shown in a movement-tracking task. While the studies mentioned above were done in single-session designs and demonstrated transient tDCS effects in within-session learning, it has also been suggested that tDCS affects offline learning and consolidation. For example, anodal tDCS of M1 enhanced the offline effect of motor learning (Reis et al., 2009), anodal tDCS of V1 blocked overnight consolidation of a visual contrast detection task (Peters et al., 2013), and anodal tDCS applied over the dorsolateral prefrontal cortex enhanced verbal working memory training (Richmond et al., 2014).

In the auditory domain, electroencephalography (EEG) measurements demonstrated that anodal tDCS applied over the left temporal cortex increased auditory-evoked potential (AEP) P50 amplitudes (Zaehle et al., 2011), indicating that the stimulation does modulate the functional response of the auditory cortex. At a behavioral level, anodal tDCS showed an enhancing effect and cathodal tDCS has a blocking effect on a pitch memory task when applied over left supramarginal gyrus (Vines et al., 2006; Schaal et al., 2013). Deterioration effects of cathodal tDCS have also been found in a pitch detection task with 2 mA of tDCS over left and right Heschl's gyri (HG), with a stronger effect on the right HG (Mathys et al., 2010), as well as in a pitch matching task with 2 mA of cathodal tDCS over inferior frontal and superior temporal cortical regions (Loui et al., 2010). On the other hand, degradation of performance with anodal tDCS applied over the right auditory cortex has been reported in a pitch discrimination task (Tang and Hammond, 2013). In this study, while anodal tDCS did not interrupt within-session learning rate, it degraded overall task performance, which persisted at least until 24 h later. An effect of cathodal tDCS was not assessed in this study.

According to the existing studies, polarity-specific effects of tDCS on auditory tasks seem to vary, such that detrimental effects on pitch manipulation tasks both from anodal tDCS and cathodal tDCS have been reported. Possible reasons for these diverse results in the literature could be the different parameters of stimulation used across studies, or the wide variety of tasks used, which are not always well-characterized in terms of the neural substrates involved. In the current study, we took advantage of a microtonal melody pitch discrimination learning task that was previously used in a study by Zatorre et al. (2012). Functional magnetic resonance imaging (fMRI) showed that the right auditory cortex was associated with learning to discriminate these patterns, because activity in this area was specifically modulated after learning. In addition, the degree to which activity in right auditory cortex tracked pitch changes prior to learning was found to predict individual differences in subsequent learning. Furthermore, there is a wealth of other evidence that the right auditory cortex is important for fine-grained pitch processing (Zatorre, 1985; Zatorre et al., 1994; Griffiths et al., 1999; Johnsrude et al., 2000; Patterson et al., 2002; Brown and Martinez, 2007; Hyde et al., 2008).

Given that the right auditory cortex is well documented to be involved in learning of fine-grained pitch patterns, the current study targeted this region with tDCS. At the same time, there is strong evidence that suggests that tDCS modulates neural plasticity at a physiological level as discussed above. Thus, we hypothesized that tDCS application over the right auditory cortex would affect fine-grained pitch discrimination, as measured using an adaptive staircase procedure; we specifically aimed to assess whether performance would be changed immediately after stimulation, implying a modulation of perceptual function, or on subsequent days of testing, implying a modulation of learning or consolidation. Since neuroimaging studies are mainly correlational, an additional goal of the present study was to test for a causal influence of tDCS over the right auditory cortex on pitch processing. Given that the importance of polarity-specific effect of tDCS in the literature, we also addressed whether there is a polarity-dependent effect of tDCS in the auditory system. However, given the variability in reported outcomes concerning this parameter, we did not make a specific prediction as to the directionality of the polarity effect on behavioral outcome. To our knowledge, this is the first study addressing a polarity-specific effect of tDCS on overnight learning consolidation in the auditory domain.

## Materials and methods

### Participants

Seventy-nine participants (thirty-nine female) were recruited from the local community. Thirty-six participants did not pass the screening test (see Procedure Section) and one withdrew due to skin irritation during the tDCS application. Forty-two participants (twenty-three female) with mean age of 22.15 ± 3.24 years old (mean ± SD) passed the screening test and completed all training. All participants had normal hearing, no neurological or psychological disorders, and no history of epilepsy. All participants had less than 6 years of formal musical training according to a self-reported history of musical experience. This study was approved by the Research Ethics Board of the Montreal Neurological Institute. All participants gave written informed consent to participate in this study. The participants were randomly divided into three groups of 14 people each: an anodal tDCS group received tDCS with the anodal electrode placed over the right temporal cortex, a cathodal tDCS group received tDCS with the cathodal electrode placed over the right temporal cortex, and a sham group received sham tDCS. The participants (but not the experimenter) were blind to the stimulation conditions.

### Procedure

**Figure 1:**
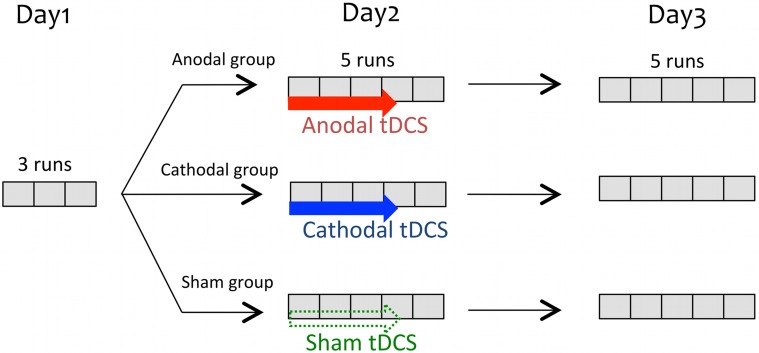
**Global procedure: Participants performed the same pitch discrimination task on three consecutive days**. The baseline performance was obtained from three runs of the task on Day1. On Day2, participants were divided into three groups and each group received either anodal, cathodal, or sham tDCS concurrent with the task. The duration of stimulation (depicted by colored arrows) was variable depending on the individual's speed, but typically encompassed the first three runs. On Day3, participants came back to perform 5 runs of the task without tDCS, which allow us to assess possible retention effect of tDCS on the task.

The training was carried out on three consecutive days. Participants performed the micromelody pitch discrimination task (see below) during several runs of testing on each day. Each session was separated by 23.97 h ± 0.25 [mean ± SD (h)]. On the first day, participants were tested with three runs of the task to measure their baseline performance, without any tDCS. This test was also used as a screening test. As we aimed to examine learning effects, participants who showed mean pitch discrimination thresholds that were already smaller than 15 cents on this test (*n* = 6) were not retained because there would be no room for improvement. Also, in order to be able to measure any deterioration effect of tDCS, participants who showed pitch thresholds that were higher than 40 cents on the Day1 test (*n* = 29) were also not retained. On the second day, participants received tDCS while they performed the same task as on Day1. Participants performed five task runs in total during and after the tDCS. On the third day, participants performed five more task runs identical to those of the other days without receiving tDCS (Figure 1).

### Stimuli

**Figure 2:**
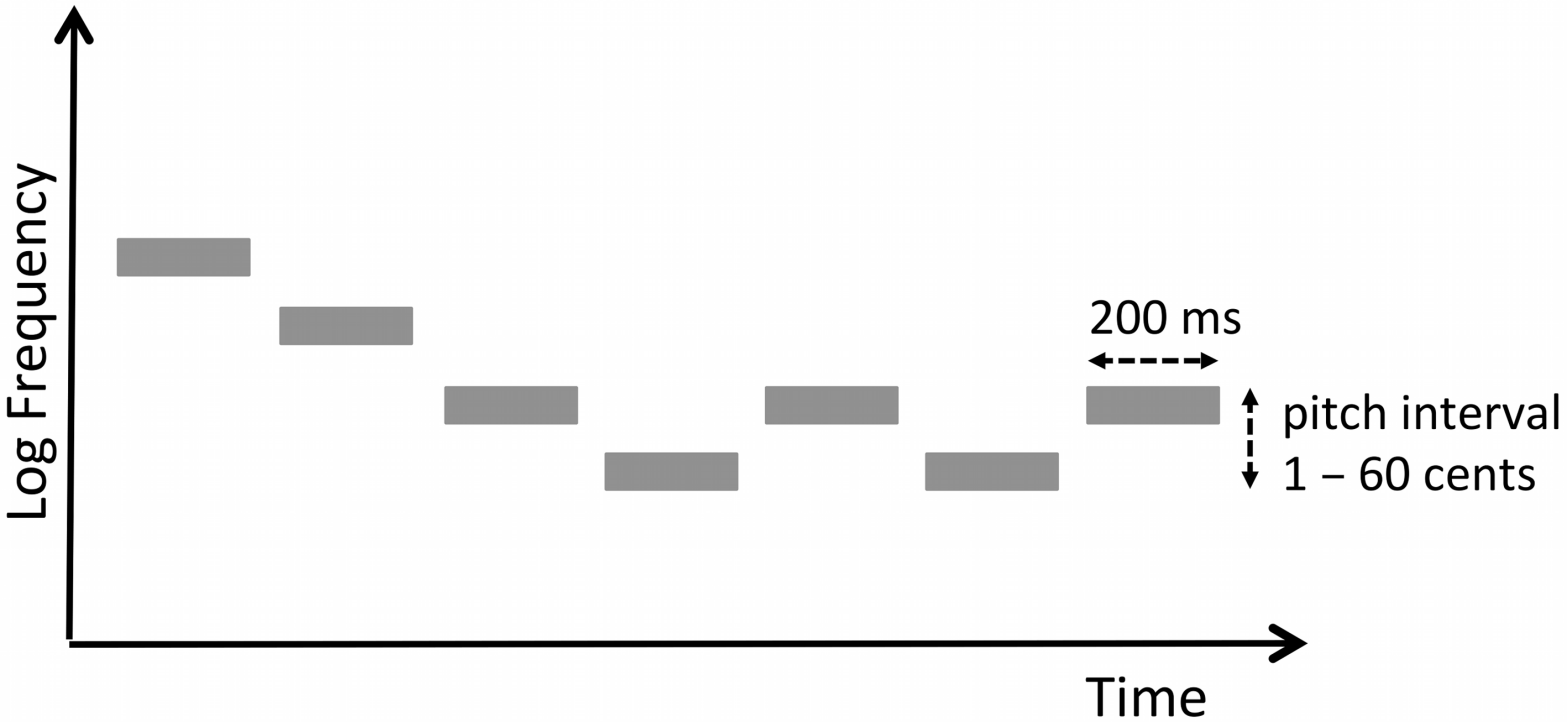
**Schema of a micromelody**. A single micromelody consisted of seven pure tones, each of 200 ms duration with either two or three melodic contour changes. The 4th tone was set to 250 Hz; the intervals between notes varied by between 1 and 60 cents (where cent refers to a logarithmic unit such that 100 cents is equivalent to one semitone in the western musical scale).

The pitch discrimination task made use of the same stimuli as those used in a previous study (Zatorre et al., 2012). We define micromelodies as tone patterns with pitch intervals that are smaller than 100 cents. A cent refers to a logarithmic frequency unit, such that 100 cents corresponds to one semitone, the smallest unit of pitch change in the Western musical scale. A single micromelody consisted of seven pure tones. Each tone was 200-ms long, with an inter-tone interval of 150 ms. Therefore, the duration of each micromelody was 2.35 s. The middle tone (i.e., the 4th tone) of each micromelody was set to the frequency of 250 Hz; all other tones varied in frequency in relation to this one with either zero or at most one consecutive repetition of any given tone, and with either two or three inversions of melodic contour (e.g., down-down-down-**up-down**-down-**up** would contain three inversions, denoted in boldface) (Figure 2). In the pitch discrimination task, there was an inter-stimulus interval of 1 s between two melodies presented in each trial.

### Task

The micromelody pitch discrimination task was administered via a computer on each day for all stimulation groups. The task consisted of a two-alternative, forced-choice procedure in which a pair of micromelodies was presented, and participants were required to answer whether the two items were the same or different by clicking on a mouse button. For half the trials, the stimuli were the same, and for the other half different. After each trial, participants received visual feedback on whether or not their response was correct. Each run consisted of two concatenated staircase procedures with a “2 down-1 up” adaptive level variation rule. The first staircase procedure was fixed in terms of number of reversals and the latter staircase procedure was fixed in terms of number of trials.

Each run began with a pair of 60-cent pitch interval melodies and participants judged whether they were the same or different. In the first staircase procedure, melodies were presented at the following intervals: 60, 50, 40, 30, 20, 10, 5, 3, or 1-cent. After two successive correct responses, the trial difficulty increased by decreasing the pitch interval, and after an incorrect response, the trial difficulty decreased by increasing the pitch interval (e.g., if a participant gave correct responses to two 60-cent trials in a row, the next trial would be a 50-cent trial.) For the first 6 participants, the reversal number was fixed at 9, and for the remaining 36 participants the reversal number was fixed at 4. We instituted this change after initial testing to save time, and because both reversal numbers were very highly correlated with the final threshold values and therefore didn't significantly change the results (Figure S1 in Supplementary Material).

**Figure 3:**
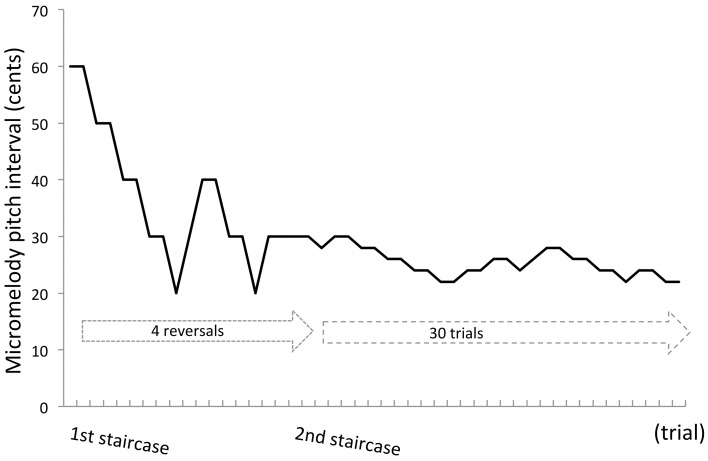
**An example of a run in the staircase procedure**. A single run of the task consisted of two concatenated staircase procedures. In the first staircase, a participant starts with 60-cent pair of stimuli and completes a fixed number of reversals, either 4 or 9. In the second staircase, continuous with the first, the task continues without change but the trial number is fixed to 30. The threshold was determined by averaging the last 30 trials. In this example, the threshold of this run was 29 cents.

After the fixed number of reversals, the second staircase procedure started continuously with the last trial of the first staircase procedure. For the second part, the difficulty level changed by 2 cents on each trial, and the trial number was fixed at 30 for all participants (Figure 3). This staircase procedure allowed us to determine a subject's threshold in a fine-grained manner.

The total task length and the total trial number varied across individuals depending on how long it took to complete the reversal number in the first part of the task. As a consequence, on Day2, 3.02 ± 0.46 runs (mean ± SD) overlapped with an application of tDCS, and the rest of the runs were performed after the application of tDCS.

### Transcranial direct current stimulation

tDCS was applied using DC-STIMULATOR (neuroConn GmbH, Ilmenau, Germany). The active electrode was placed over the right temporal cortex and the reference electrode was placed above the left eyebrow. The active electrode was anodal for the anode group, and cathodal for the cathode group. We used the scalp location closest to the lateral edge of HG as a landmark to place the active electrode. The location of the right HG was determined using a neuronavigation system, Brainsight (Rogue Research Inc., Montreal, Canada) with a single brain scan as a template. Each electrode was 35 cm^2^ in surface area and covered by a saline-soaked sponge. Stimulation was delivered for 20 min with 8 s of ramp-up and ramp-down phases at the beginning and the end. The current intensity was set to 1 mA for all stimulation conditions. For the sham stimulation, the current was ramped up to 1 mA as in the active conditions, but was only kept on for 30 s, and then ramped down.

## Results

The dependent variable used was the threshold value, that is, the smallest micromelody pitch interval, in cents, that could be discriminated during a run. For all groups, the last 30 trials (i.e., the trials in the second staircase procedure) of each run were used for analysis. In the following analyses, the upper 7.5% and the lower 7.5% of the trials were removed from all trials collected across runs from each participant in order to reduce any effect of outliers and thereby allow parametric statistics to be employed. The average of the thresholds of the three blocks on Day1 was used as a baseline value. The baseline thresholds were compared across groups using one-way analysis of variance (ANOVA) and were not significantly different [*F*_(2, 39)_ = 0.15, *p* = 0.86], indicating that the random assignment was successful in producing comparable groups prior to stimulation.

**Figure 4:**
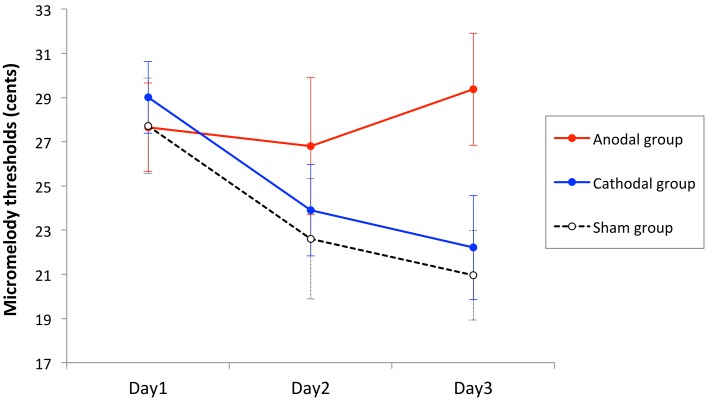
**Mean performance as a function of time for the three stimulation groups**. Each point represents the mean threshold of all runs in 1 day for the anodal, cathodal, and sham groups. The error bars are standard error of the mean. The anodal tDCS group did not show significant learning effect, whereas the cathodal and sham tDCS significantly improved over time.

Thresholds of all blocks in a day for each individual were averaged to represent a threshold for that day. The thresholds were analyzed using two-way mixed design analysis of variance (ANOVA) with stimulation group as between-subject variable (anodal, cathodal, and sham), and time as within-subject variable (Day1, Day2, and Day3). There was a significant main effect of time [*F*_(2, 78)_ = 5.19, *p* = 0.008], but no main effect of stimulation group [*F*_(2, 39)_ = 1.29, *p* = 0.29], and a marginal interaction effect between time and stimulation group [*F*_(4, 78)_ = 2.13, *p* = 0.085]. However, the linear trend component of this analysis did show a significant interaction effect between time and stimulation group [*F*_(2, 39)_ = 4.20, *p* = 0.02], indicating that the learning rate differed across groups (Figure 4). To examine this effect further, we computed learning slopes for each individual. The slope was calculated individually by computing a linear trend line using ordinal least square, and a goodness of fit was assessed with R-square. The mean R-square values were 0.43 ± 0.39, 0.48 ± 0.29, 0.60 ± 0.31 (mean ± SD) for the anodal tDCS group, cathodal tDCS group, and sham tDCS group, respectively. The average slope values were 0.86 ± 4.30, −3.40 ± 4.31, and −2.48 ± 5.37 (mean ± SD) for anodal tDCS group, cathodal tDCS group, and sham tDCS group, respectively. The slope values were compared across stimulation groups by One-Way ANOVA and there was a significant main effect [*F*_(2, 39)_ = 3.2, *p* = 0.05]. A *post-hoc* Tukey's HSD test showed a significant difference between the anodal and cathodal groups (*p* = 0.05) and a trend toward significant difference between the anodal and sham groups (*p* = 0.16), but no significant difference between the cathodal and sham groups (*p* = 0.87).

In addition, a score reflecting the improvement rate of each individual was also calculated using an average threshold of each day as follows:

$$Total\;improvement\left(\%\right)=\frac{\left(Day1\;threshold-\;Day3\;threshold\right)}{Day1\;threshold}\times 100$$

**Figure 5:**
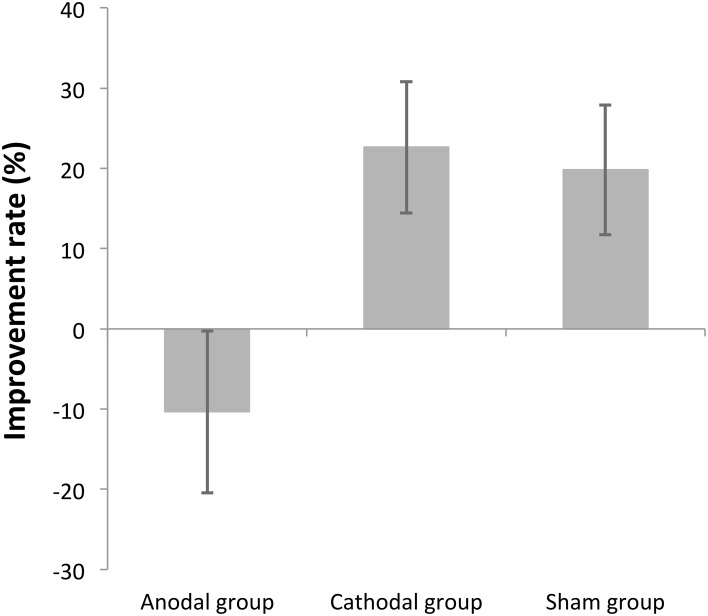
**Task performance changes in each group as percentage of improvement**. The overall improvement rates were calculated by comparing Day3 threshold to Day1 threshold in each participant, expressed as a percentage, and then averaged across participants in each stimulation group. The error bars are standard error of the mean. The cathodal and the sham groups significantly improved over 3 days but the anodal group did not improve.

This analysis rules out individual variability of the baseline, as it normalizes for initial performance. The average improvement rate was −10.35% ± 37.77, 22.65% ± 30.67, 19.83% ± 30.26 (mean ± SD), for the anodal, cathodal, and sham stimulation groups, respectively. The improvement rate was compared among stimulation groups by One-Way ANOVA. There was a significant difference across groups [*F*_(2, 39)_ = 4.3 *p* = 0.02]. Subsequent pairwise comparison tests, conducted using Tukey's HSD, indicated that there were significant differences between the anodal and the cathodal groups (*p* = 0.03), as well as between the anodal and the sham groups (*p* = 0.05), but there was no significant difference between the cathodal and sham groups (*p* = 0.97) (Figure 5).

Besides an overall effect of tDCS across days, we were also interested in whether there was any difference when comparing pitch discrimination thresholds during tDCS and immediately after tDCS on Day2. The five blocks on Day2 were therefore divided into “during tDCS” and “immediately after tDCS” individually; blocks on “during tDCS” were performed with 20 min of tDCS and “immediately after tDCS” were performed after the termination of tDCS. For sham tDCS group, blocks that were performed in the first 20 min were categorized as “during tDCS.” 3.02 ± 0.46 (mean ± SD) blocks were overlapped with tDCS.

In order to directly assess any difference between “during tDCS” and “immediately after tDCS,” thresholds of those times were compared separately with paired-sample *t*-test for each stimulation group. None of the groups showed significant difference in thresholds between “during tDCS” and “immediately after tDCS” {anodal tDCS group [*t*_(13)_ = 0.23, *p* = 0.82], cathodal tDCS group [*t*_(13)_ = 1.15, *p* = 0.27], sham tDCS group [*t*_(13)_ = 0.12, *p* = 0.91]}.

## Discussion

In this study, we demonstrated that anodal tDCS over the right temporal cortex interrupted pitch discrimination learning, whereas cathodal tDCS did not affect learning compared to sham tDCS. A previous study (Zatorre et al., 2012) using the identical stimuli and task (but tested over six sessions spread out over 2 weeks instead of 3 days) had demonstrated that brain activity in the right auditory cortex was modulated after learning; specifically, it showed that the slope of blood oxygenation signal as a function of pitch interval size decreased after learning, suggesting that pitch information was better encoded after training. The present findings extend these data by showing a causal effect of stimulation on the right temporal cortex, supporting its role in encoding fine-grained pitch information.

The fact that the sham and cathodal tDCS groups showed significant improvement on the task over 3 days confirms that the training procedure was effective. This finding is in line with other studies that have shown auditory learning within a short term (Bosnyak et al., 2007; van Wassenhove and Nagarajan, 2007). An electrode montage of the anodal electrode on the right temporal cortex and the cathodal electrode on the left supraorbital region has been shown to modulate auditory evoked potentials (Zaehle et al., 2011), supporting the conclusion that the electrode montage in our study is valid to modulate neural activity in the auditory cortex. The principal finding that the cathodal and sham groups showed improvement but the anodal group did not improve or degrade was observed regardless of statistical approaches applied, indicating that the finding is consistent across statistical analyses and confirming the polarity-specific effect of tDCS on pitch discrimination learning. Since there was no difference in the comparison of “during tDCS” and “immediately after tDCS” on Day2, we have no evidence of a perceptual change due to tDCS application, at least under the conditions used in the present study.

### Anodal stimulation blocked auditory learning but not perception

The direction of the anodal tDCS effect on our pitch discrimination task seems to be consistent with a previous study using a pitch task by Tang and Hammond (2013), where tDCS effects on pitch learning were addressed by applying anodal tDCS over the temporal cortex. The task in Tang and Hammond's study was designed for a rapid within-session learning, which was observed to be unaffected in both anodal and sham stimulation groups while overall task performance in the anodal stimulation group was degraded compared to the sham group. More importantly, these authors also found that the pitch discrimination ability of the anodal group degraded on the following day compared to the last task block of the stimulation day, suggesting a blocking effect of anodal tDCS on the consolidation of learning. This finding is also in line with our result showing that tDCS affects the consolidation of learning rather than online learning.

On the other hand, an immediate effect of tDCS on pitch discrimination has been reported by Mathys et al. (2010), where 2 mA tDCS was applied over the left and right temporal cortices. The authors reported that cathodal tDCS interfered with performance on a pitch direction discrimination task, whereas anodal tDCS did not have any effect compared to sham. These behavioral outcomes are inconsistent with our findings, which could be related to differences of timing of tDCS application and task, the current intensity, the electrode size and the task itself. First, while Mathys et al. (2010) applied tDCS only before the task, in our study tDCS was applied while participants were performing the task. This difference in timing of tDCS application seems to be crucial according to previous studies that showed that only concurrent application of tDCS and task enhanced task learning or increased cortical excitability (Stagg et al., 2011; Antal et al., 2014; Gill et al., 2014; Reis et al., 2015). In fact, it is suggested that tDCS preferentially modulates the neural substrate that is recruited by an ongoing activity, a phenomenon referred to as activity-selectivity of tDCS (Reato et al., 2010; Reis and Fritsch, 2011; Ranieri et al., 2012; for a review Bikson and Rahman, 2013). Interestingly, Bortoletto et al. (2014) demonstrated the commonly-expected polarity-specific effect can be even reversed by an ongoing motor task that also modulates cortical excitability. Second, non-linear effects of current intensity have been reported in motor areas: measurement of MEP revealed that cathodal tDCS at 2 mA over M1 increased cortical excitability while cathodal stimulation at 1 mA decreased it (Batsikadze et al., 2013). It is possible that there is a non-linear effect of current intensity or current density in auditory areas, too. In fact, due to the combination of the higher current intensity and the smaller electrode (i.e., 2 mA/16.5 cm^2^), even higher current density was applied in Mathys et al. (2010) study, compared to that of ours (i.e., 1 mA/35cm^2^). Such differences could result in different directions of polarity-specific effects at a behavioral level.

### Contributing factors to effects of tDCS

Although anodal stimulation has been known to increase cortical excitability, as shown by many studies in the past (Bindman et al., 1964; Purpura and McMurtry, 1965; Nitsche and Paulus, 2000, 2001; Baudewig et al., 2001; Antal et al., 2003) and positive effects with anodal stimulation have been reported at a behavioral level (Antal et al., 2004; Boggio et al., 2006; Flöel et al., 2008; Reinhart and Woodman, 2015), it is also known that polarity is not the only factor that determines the effect of tDCS application.

In fact, the direction of polarity-specific tDCS effect on multiple-day learning in healthy participants differs among different domains: enhancing effects of anodal tDCS have been reported in the motor domain (Reis et al., 2009) and verbal working memory (Richmond et al., 2014), while blocking effects have also been reported in visual (Peters et al., 2013) and auditory domains (Tang and Hammond, 2013), as well as in the present experiment. One reason for this variability could be that the electrical stimulation is manifested in different ways at the cellular level, depending on properties of neurons that are affected, such as location, orientation, and shape. For example, although anodal stimulation typically causes depolarization, it can also cause hyperpolarization of neurons that are located superficially to cortex (Purpura and McMurtry, 1965). In addition, electrical stimulation has different effects on different classes of cells, such as pyramidal cells and interneurons, with the former being more sensitive (Radman et al., 2009). Finally, a current applied parallel to a neuron can cause hyperpolarization, but a current applied perpendicularly can have no effect or the opposite effect (Jefferys, 1981; Bikson et al., 2004; Kabakov et al., 2012). In our study, we placed an active electrode over the right HG, where the cortex is folded and neural orientations are not uniform. Therefore, predicting the net effect of tDCS on neurons in temporal cortex and the related network is challenging at both a physiological and a behavioral levels. Moreover, computational models of electric field propagation suggest that a gyrus can be affected by a high magnitude of electric field according to how the cerebrospinal fluid, which is the most conductive tissue under the skull, flows into gyri (Wagner et al., 2007; Datta et al., 2009). This anatomical property adds an additional complexity for predicting tDCS effects.

### Learning and tDCS

Regardless of the direction of effect from anodal tDCS, it seems to be consistent that tDCS has an influence on task learning and its consolidation across domains, such as motor, visuomotor, visual, language, and memory (Antal et al., 2004; Flöel et al., 2008; Reis et al., 2009; Peters et al., 2013; Richmond et al., 2014). These behavioral effects are supported by MRS studies that confirmed changes in concentrations of neurotransmitters and their receptors during and after tDCS application: anodal tDCS reduces GABA (Nitsche et al., 2004; Stagg et al., 2009, 2011) and cathodal tDCS reduces glutamate (Nitsche et al., 2004; Stagg et al., 2009). Pharmacological studies also suggested that the after-effect of tDCS is NMDA-dependent (Liebetanz et al., 2002; Nitsche et al., 2003b; Monte-Silva et al., 2013). This evidence suggests that the tDCS mechanism involves an LTP-like effect for learning. In the auditory domain, however, there is no report so far that assessed changes in neurotransmitter levels during/after tDCS of auditory cortex. Yet it is possible that the mechanism of how tDCS affects learning is shared among domains. Although the alteration of neurotransmitter levels may not be the only underlying factor of the behavioral change—in fact, homeostatic plasticity and metaplasticity also seem to be relevant to tDCS effects (Siebner et al., 2004; Lang et al., 2007; Nitsche et al., 2007; Kuo et al., 2008; Stagg et al., 2011)—the physiological effects of tDCS in the auditory learning will need to be addressed further in the future.

### Limitations

In order to account for other cognitive factors that could affect task performance, such as working memory and attention, it will be necessary in the future to add a control task condition to assess task specificity. We would predict that a learning task not based on fine-grained pitch differences would not be affected by tDCS applied over the right auditory cortex. The previous fMRI study by Zatorre et al. (2012) demonstrated that the right auditory cortex was modulated in the micromelody pitch discrimination task, and this study strongly supports our assumption that the placement of anodal electrode over the right temporal cortex was effective to stimulate right auditory cortex, and hence that the observed behavioral change was due to interference with the function of the right auditory and surrounding areas. At the same time, considering the large electrode size (5 × 7cm) relative to the size of auditory cortex and past modeling studies that have shown tDCS current has diffuse effect (Datta et al., 2009; Bikson et al., 2012; Miranda et al., 2013; Ruffini et al., 2014), we do not presume that the stimulation affected specifically only the right auditory cortex. Future studies will also have to compare stimulation of right auditory cortex with other control regions to determine the regional specificity of the effect; in particular, comparison of right and left stimulation sites will be important to confirm the hemispheric differences predicted from the fMRI literature. Finally, another limitation to keep in mind is that several brain imaging studies have shown that tDCS can also influence remote brain regions beyond the stimulation site (Lang et al., 2005; Kwon et al., 2008). Such limitations apply also to transcranial magnetic stimulation, which when applied over the right HG caused remote effects on brain activity on the contralateral homolog left HG (Andoh and Zatorre, 2013). Thus, future experiments will benefit not only from control conditions and stimulation sites, but may also need to document the changes in activity patterns induced by tDCS using other methods, such as fMRI and EEG for example.

## Conclusion

The present study showed that anodal tDCS applied over the right auditory cortex has a causal effect on auditory pitch discrimination learning. As opposed to many studies showing enhancing effect of tDCS, our results showed a blocking effect of anodal tDCS applied over rHG on pitch learning, suggesting that tDCS effects are not identical across domains at a behavioral level. Although tDCS is often proposed as a tool for neural enhancement, a better understanding of its underlying mechanism is still needed before further application. Further research will be necessary to establish the site and task specify of the effect. In addition, the stimulation parameters and details of task and stimulation application have to be carefully considered.

### Conflict of interest statement

The authors declare that the research was conducted in the absence of any commercial or financial relationships that could be construed as a potential conflict of interest.

## Supplementary material

Figure S1 **Comparison between runs with 9 reversals and runs with 4 reversals**. The correlation between the average threshold, which is used in the analysis, and the pitch discrimination difficulty reached at the last trial of the first staircase was assessed. The correlations were highly significant in both groups. Therefore, whether the number of reversals is 9 or 4 in the first staircase does not make a significant difference in the final threshold.
