# Supplementary figures and images for: Polarity-specific transcranial direct current stimulation disrupts auditory pitch learning
Source: Front Neurosci. 2015 May 18;9:174. doi: 10.3389/fnins.2015.00174 (PMC4434966; doi:10.3389/fnins.2015.00174)

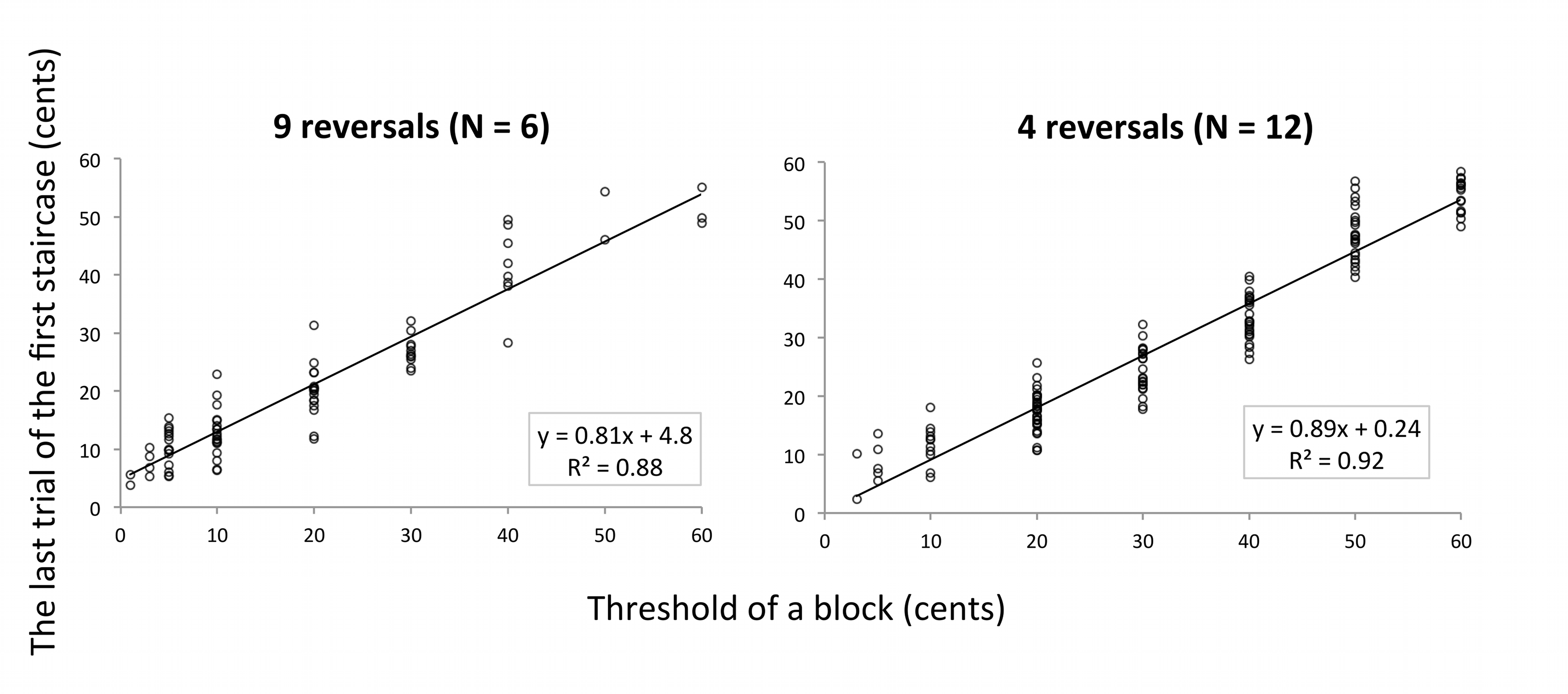

Supplement: Figure S1 — Comparison between runs with 9 reversals and runs with 4 reversals. The correlation between the average threshold, which is used in the analysis, and the pitch discrimination difficulty reached at the last trial of the first staircase was assessed. The correlations were highly significant in both groups. Therefore, whether the number of reversals is 9 or 4 in the first staircase does not make a significant difference in the final threshold. [file Image1.TIFF]
